# Supplementary material for: When a Pandemic Strikes: Resilience of Swedish Academics in the Face of Coronavirus
Source: Int J Environ Res Public Health. 2022 Oct 16;19(20):13346. doi: 10.3390/ijerph192013346 (PMC9602465; doi:10.3390/ijerph192013346)
Supplement: Supplementary file 1 [file ijerph-19-13346-s001.zip › ijerph-1924094-supplementary.pdf]

Coping with Covid-19

We want to ask you if you can take part in a study on the coping methods in relation to the Covid-19.

Purpose:

The following questions relate to the way you have dealt with the situation during the Covid-19 (Corona virus), which was declared a pandemic by the World Health Organization (WHO) in March 2020. Countries have begun to take precautions to prevent the spread of the virus, and these precautions have affected individuals and society on al levels. There are many ways to deal with problems. Of course, different people deal with their problems in different ways, and we are interested in how you have dealt with the situation, especially the ones related to your emotions such as worries, fear, and loneliness, which were caused by the spread of the virus in your country and the world. So in this questionnaire, wherever we mention "situation", we refer to the situation caused by the spreading of the Covid-19 virus, which is characterized by a lack of physical contact with people in general, working at a distance from your regular office, an extreme concern of yours and your family members' health, concerns about the economic situation for you personally and for others, both now and for the future, as well as general concerns for the future.

About the study:

Each question says something about a particular way of dealing with the situation; these ways are called coping methods. We want to know to what extent you have used these coping methods. Please use response options. In this questionnaire whenever we use the word spirituality, we refer also to that kind of spirituality, which is NOT necessarily related to religiosity.

The questions deal with your experiences and shouldn't result in any uncomfortable situation or risks for you. As a research team, we have long experience of coping studies as well as survey studies. Your answers to the survey will be saved at the repository of (anonymized), which is password protected. Only the researchers have access to the material, which is preserved for 10 years. We follow the GDPR directives (EU 2016/679). We will present the results of the study on group and thematic levels only, in scientific publications. If you want more information about the study and its results, you can contact the project leader (anonymized) or any of the other researchers mentioned here.

Consent

I have been informed about the study “Coping with Covid-19” and I give consent to participate in this study:
Yes ❑

Coping instrument

What do / did you do to handle the situation during the “COV-19 time”, if you feel / felt stressed, sad or depressed or other reactions?

	Never – 0	Sometimes -1	Quite often - 2	Very often- 3
**27.** Have you thought that your life is part of a greater whole?	❑0	❑1	❑2	❑3
**27.** Have you thought or felt that a spiritual force exists in you to help you deal with the situation?	❑0	❑1	❑2	❑3
**27.** Has nature been an important resource for you in how to deal with your stress/sadness or other negative feelings?	❑0	❑1	❑2	❑3
**27.** Has being alone and having the chance to contemplate helped you deal with the situation?	❑0	❑1	❑2	❑3
**27.** Have you listened to the sounds of the surrounding nature?	❑0	❑1	❑2	❑3
27. Have you walked or engaged in any activities outdoors that give / gave you a spiritual feeling?	❑0	❑1	❑2	❑3
27. Have you regularly meditated dealing with your stress/sadness or other negative feelings?	❑0	❑1	❑2	❑3
27. Have you sought spiritual help from a religious leader?	❑0	❑1	❑2	❑3
27. Have you thought that the COV-19 was caused by an evil power?	❑0	❑1	❑2	❑3
27. Have you wondered if God has left you or become angry that God is not present to help you?	❑0	❑1	❑2	❑3
27. Have you had the feeling of a strong connection with God?	❑0	❑1	❑2	❑3
27. Have you visited the church, synagogue,	❑0	❑1	❑2	❑3

mosque, temple, or to other religious places?				
27. Have you prayed to God or other religious figure to make things better?	❑0	❑1	❑2	❑3
27. Have you listened to religious or spiritual music?	❑0	❑1	❑2	❑3
27. Do / did you think that you have done your best and now it is only God who is in control?	❑0	❑1	❑2	❑3
27. Have you tried to get control of your situation directly without the help of God or other religious figure?	❑0	❑1	❑2	❑3
27. Do you believe in God or other religious figure?	❑0	❑1	❑2	❑3
27. Do you think there is a higher power or giving power?	❑0	❑1	❑2	❑3
27. Would you say that you come from a religious family?	❑0	❑1	❑2	❑3

Meaning, resilience, and general health

27. Please, rate how much the following help you to make sense/give your life meaning during the COV-19?

	Doesn't help at all -1	Helps somewhat -2	Helps often -3	Helps very much -4
A. Family				
B. Friends				
C. Religion/Spirituality				
D. Work/School				
E. Other: (specify) ……………………				

*For each item, please mark what best indicates how much you agree with the statements as they apply to you over the last **month**.*

		not true at all (0)	rarely true (1)	sometimes true (2)	often true (3)	true nearly all the time (4)
27. A	I am able to adapt when changes occur.	☐0	☐1	☐2	☐3	☐4
27. B	I tend to bounce back after illness, injury, or other hardships.	☐0	☐1	☐2	☐3	☐4

27. In general, would you say your health is:

Poor ❑1 Fair ❑2 Good ❑3 Very good ❑4 Excellent ❑5

Occupational health

27. Do you work more than you are contracted each week if you started to work at home as a result of the COVID-19 situation?

No ❑0 Yes ❑1

27. How satisfied are you with your current work from home arrangement?

❑1 Very dissatisfied
❑2 Somewhat dissatisfied
❑3 Neither satisfied nor dissatisfied
❑4 Somewhat satisfied
❑5 Very satisfied

26. If you have challenges to work from home how do you cope with them?

	Never	Seldom	Sometimes	Often	Always
A. I have social contact with my colleagues/classmates through distance tools and other social media	❑1	❑2	❑3	❑4	❑5
B. I have social contact with my family and friends through distance tools and social media	❑1	❑2	❑3	❑4	❑5
C. I think about what I can do, rather	❑1	❑2	❑3	❑4	❑5

than what I can't					
D. I make myself busy with my working day because it makes me feel useful	❑1	❑2	❑3	❑4	❑5
E. I read/collect information from Public health authorities in my country or World Health Organisation and keep myself update with public health news	❑1	❑2	❑3	❑4	❑5
F. I trust state or local health authorities in my country	❑1	❑2	❑3	❑4	❑5
G. I give myself a news time limit for each day	❑1	❑2	❑3	❑4	❑5
H. I avoid recommendations that are not from public health authorities in my county or from World Health Organisation.	❑1	❑2	❑3	❑4	❑5
I. I care for my mental and physical health	❑1	❑2	❑3	❑4	❑5
J. I try providing kindness and support to the people around me	❑1	❑2	❑3	❑4	❑5
K. I make sure to have access to medical resources and the health services if I need to seek health care	❑1	❑2	❑3	❑4	❑5
L. I believe we are all in this together, and with solidarity we can find the best solutions for handling covid-19	❑1	❑2	❑3	❑4	❑5

Background questions

We end by some background information, which are needed for our analysis.

27. What is your current employment/student status?

1❑ Employed full-time
2❑ Employed part-time

3❑ Campus student

4❑ Distance learning student

28. Which year were you born? …….

29. What is your gender?

1❑ Man
2❑ Woman

3❑ None of the above

30. What is your highest education?

1❑ Lower than elementary school

2❑ Elementary school or equivalent

3❑ Gymnasium or equivalent

4❑ University or equivalent

31. What is your current relationship status?

1❑ Married
2❑ Divorced
3❑ Engaged
4❑ Widowed
5❑ Single
6❑ Other (please briefly specify)………………

32. Do you have children?

❑ 0 Yes

❑ 1 No

33. What characterizes the place you live?

❑ 0 Capital

❑ 1Mid-large city, not capital
❑ 2 Small town close to a large city
❑3 Small town far from a large city

34. What is your country of birth? (please write)...

35. Country of residence: (please write) ……………………………..
